# Metabolomic Analysis of Exosomes Derived from Lung Cancer Cell Line H460 Treated with SH003 and Docetaxel

**DOI:** 10.3390/metabo12111037

**Published:** 2022-10-28

**Authors:** Yu-Jeong Choi, Kangwook Lee, Miso Jeong, Yong Cheol Shin, Seong-Gyu Ko

**Affiliations:** 1 Department of Science in Korean Medicine, Graduate School, Kyung Hee University, Seoul 02447, Korea; 2 Department of Preventive Medicine, College of Korean Medicine, Kyung Hee University, Seoul 02447, Korea

**Keywords:** non-small cell lung cancer, exosome, SH003, docetaxel, pyrimidine metabolism, metabolism of xenobiotics by cytochrome P450, predictive biomarker

## Abstract

Exosomes released from tumor cells treated with cancer-targeting drugs reflect altered metabolic processes within the cells. Therefore, metabolites in exosomes can be used as markers to predict the therapeutic response or identify therapeutic targets. In this study, metabolite changes in exosomes were investigated by co-administration of the herbal extract SH003 and docetaxel (DTX), which exert a synergistic anti-cancer effect on lung cancer cells. Exosomes released from cells treated with SH003 and DTX were purified, and untargeted metabolic profiling was performed by liquid chromatography–tandem mass spectrometry. Analysis of altered metabolic-based pathways showed that the combined treatment synergistically increased pyrimidine metabolism compared with single-drug treatment. Additionally, xenobiotic metabolism by cytochrome P450 was specifically increased in cells treated with the combination. However, the released exosomes and increased metabolites in exosomes did not affect the anti-cancer effect of SH003 and DTX. Therefore, our study suggests that metabolite profiling can be used to evaluate the efficacy of combined treatments. Furthermore, such exosome-based metabolism may facilitate understanding the physiological endpoints of combination therapy in human biofluids.

## 1. Introduction

Exosomes are small membrane vesicles (30–150 nm) released by several cell types and are stable sources of cell-derived genetic materials that modulate multiple signaling pathways in recipient cells [1]. Cancer-derived exosomes are regarded as major mediators of tumor progression, metastasis, multidrug resistance, and immune modulation [2]. Moreover, cancer exosomes contain potential cancer-related RNAs, DNAs, and proteins and are stable in body fluids, including blood, saliva, and urine, suggesting that exosomes can be used as diagnostic and prognostic biomarkers of cancer [3]. Therefore, exosomes have been proposed to be potential therapeutic, diagnostic, and prognostic markers of several cancers [4].

Liquid chromatography–mass spectrometry (LC-MS)-based metabolomics, which is the large-scale profiling of metabolites in biofluids, cells, and tissues, is an unbiased tool for biomarker discovery. Currently, metabolomes of exosomes have gained interest in cancer research [5,6,7]. Numerous studies have reported the results of analyzing metabolomic alterations in cancer cells in response to anti-cancer drugs, which is helpful in understanding the mechanisms of anti-cancer drug actions and finding potential biomarkers for cancer diagnosis [8,9,10,11,12,13]. Interestingly, metabolites in cancer cell-derived exosomes are involved in cancer progression and multidrug resistance, suggesting that unbiased metabolomic study of cancer cell-derived exosomes is essential to identify novel cancer biomarkers for prognosis, prediction, and therapeutic responses.

SH003 is a novel herbal mixture for the treatment of several cancers including non-small cell lung cancer (NSCLC) [14,15,16,17,18,19,20,21,22,23,24,25]. The molecular mechanism of SH003 in cancer inhibition is associated with both the induction of cell cycle arrest, apoptosis, and autophagy and the suppression of tumor angiogenesis [26]. Recently, we demonstrated that SH003 is a good partner to combine with docetaxel, a conventional chemotherapy medication, for the treatment of NSCLC patients [15]. Combined treatment with SH003 and docetaxel synergistically decreases the growth of NSCLC cell lines in vitro and in vivo. Moreover, the SH003-docetaxel combination strategy exerts an anti-cancer effect by inhibiting the EGFR/STAT3 signaling pathway. Based on these results, we have progressed to phases I and II in clinical trials for SH003–docetaxel combination therapy of NSCLC in South Korea [27]. However, detailed insights into the complex anti-cancer mechanisms of the SH003–docetaxel combination therapy still need to be explored.

Here, we analyzed metabolic alterations in the exosomes of lung cancer cell line H460 treated with SH003 and docetaxel to explore potential anti-cancer mechanisms and biomarkers. Isolated exosomes from H460 cell lines treated with SH003, docetaxel, or both were applied to LC-MS-based metabolomics. Our results suggested that the combined treatment significantly regulated “Pyrimidine metabolism” and “Metabolism of xenobiotics by cytochrome P450”. Moreover, treatment with SH003 and docetaxel modulated the levels of uridine and 4-(methylnitrosoamino)-1-(3-pyridinyl)-1-butanone, metabolites that may be biomarkers to predict the anti-cancer efficacy of SH003 and docetaxel combination therapy.

## 2. Materials and Methods

### 2.1. Cell Culture and Cell Viability Assay

The human lung cancer cell line H460 was obtained from the Korean Cell Line Bank (Seoul, Korea). Cells were cultured in RPMI-1640 medium (WelGENE, Daegu, Korea) supplemented with 10% heat-inactivated fetal bovine serum (JR Scientific, Inc., Woodland, CA, USA) and 1% penicillin/streptomycin solution (WelGENE), and maintained in an incubator with 5% CO_2_ at 37 °C.

Cells were seeded in a 96-well plate and treated with SH003 (300 μg/mL), DTX (1 nM), or both for 24 h that has a synergistic effect [15]. Uridine (Alfa Aesar, MA, USA) and 4-(methylnitrosoamino)-1-(3-pyridinyl)-1-butanone (NNAL; Sigma, St. Louis, MI, USA) were dissolved in D.W. and applied at the indicated concentrations. Cell viability was analyzed using a WST solution (Daeillab, Korea). Absorbance was measured by an ELISA reader (Molecular Devices, San Jose, CA, USA).

### 2.2. Isolation of Exosomes

Cells were treated with SH003, DTX, or both for 24 h. After the treatments, culture media were harvested and centrifuged at 2000× *g* for 30 min. The supernatant was collected, and exosomes were isolated using a Total exosome isolated kit (Invitrogen, Waltham, MA, USA) in accordance with the manufacturer’s instructions. In brief, the supernatant was mixed with the reagent at a 2:1 ratio and incubated at 4 °C overnight. After centrifugation at 10,000× *g* for 1 h at 4 °C, pellets were washed with 1× PBS and then recentrifuged at 10,000× *g* for 1 h at 4 °C. Exosomes were suspended in 1× PBS and stored at −80 °C until analysis.

### 2.3. Nanoparticle Tracking Analysis (NTA)

In brief, isolated exosomes were diluted at 1/5000 with PBS and the instrument was equipped with a 488-nm laser. The particles were captured at 23 °C and the average value counted per frame was approximately 80–300 particles. The exosome diameter (nm) and concentration (particles/mL) were analyzed using a Zetaview software version 8.05.12 (Particle Metrix GmbH, Inning am Ammersee, Germany).

### 2.4. Scanning Electron Microscopy (SEM)

Exosomes were fixed with 2.5% glutaraldehyde in PBS for 1 h at RT, washed with PBS at 10,000× *g* for 1 min, and then incubated with 1% osmium in D.W. for 90 min. After washing with PBS, exosomes were dehydrated in 30%, 50%, 70%, 80%, and 95% (vol/vol) ethanol solutions for 10 min and then incubated in 100% (vol/vol) ethanol for 15 min twice. For sample drying, exosomes were transferred to a 1:2 of hexamethyldisilazane (HMDS):100% ethanol for 20 min and then a 2:1 solution of HMDS:100% ethanol for 20 min. Samples diluted with 100% HMDS were coated on glass coverslips precoated in a 10% poly-L-lysine solution in PBS for 30 min at RT overnight. For non-conductive sample analysis, a thin layer of Pt was placed in the sample-processing chambers. Images were obtained using Field Emission S-4700 scanning electron microscope (Hitachi, Japan) and analyzed under the following conditions: 10 kV accelerating voltage, 10.5 μA emission current, and 9.4 mm working distance.

### 2.5. Western Blotting

Exosome pellets were suspended with 1× PBS and lysed with RIPA buffer containing protease and phosphatase inhibitors. Proteins were diluted with 5× sample buffer, separated by SDS-PAGE, and transferred to a nitrocellulose membrane. The membranes were blocked with PBS containing 0.1% Tween-20 and 5% skim milk and then incubated with primary antibodies at 4 °C overnight. The blots were incubated with horseradish peroxidase-conjugated secondary antibodies and detected using an EZ-western detection kit (Dogen-Bio, Seoul, Korea). Anti-Alix, -CD9, and -GAPDH antibodies were purchased from Cell Signaling (Danvers, MA, USA).

### 2.6. LC-MS/MS Analysis

Untargeted metabolomic analysis was performed by LC-MS/MS using an Agilent LC-MS 6550 Q-TOF instrument (Agilent, Santa Clara, CA, USA) equipped with an Agilent Infinity 1290 UPLC system (Agilent). Metabolites in exosomes were first extracted with prechilled lysis/extraction buffer (acetonitrile:water = 19:1) containing three internal standards including [3-methyl-13C]-caffeine, [13C5, 15N]-L-methionine, and [dimethyl-D6]–N, N-diethyl-M-toluamide at a 1:2 ratio and were then injected in 3 μL onto a Hypersil Gold aQ C18 column (2.1 × 100 mm, 1.9 μm) (Thermo Fisher Scientific, Waltham, MA, USA). The analytical column and autosampler were maintained at 45 °C and 4 °C, respectively. The mobile phase consisted of HPLC-grade water (JT-Baker, USA) (A) and HPLC-grade acetonitrile (JT-Baker) both containing 0.1% formic acid (Sigma). The gradient compositions were as follows: 5% B (0.0–1.0 min); 45% B (1.0–9.0 min); 90% B (9.0–12.0 min); 90% B (12.0–13.5 min); and 5% B (13.5–13.6 min). The flow rate was 0.4 mL/min with a total running time of 15 min. Electrospray ionization was used as the ionization mode. Data were collected in positive ion mode with a detection range of m/z from 50 to 1000.

### 2.7. Quantification of Exosomal Metabolites and Statistical Analysis

Data were extracted using apLCMS and characterized by xMSanalyzer software by filtering the sample at a coefficient of variation (CV) < 50%. The intensity of a metabolite was averaged, log2 transformed, normalized, and indicated by autoscaling. Hierarchical cluster analysis and principal component analysis were performed to identify differences between groups. Significant metabolites were detected by a Manhattan plot, which is indicated by −logP vs. m/z (mass-to-charge ratio), using the *p*-value. The pathway was identified by matching in the Kyoto Encyclopedia of Genes and Genomes (KEGG) database using MetaboAnalyst 5.0.

Statistical analysis was performed using PRISM 8.0.2 (GraphPad, San Diego, CA, USA). Differences in means between groups were analyzed by one-way ANOVA using Tukey’s multiple comparisons test. *p* < 0.05 indicated a statistically significant difference. Results are represented as the mean ± standard deviation.

## 3. Results

### 3.1. Characterization of Exosomes Released from H460 Cells Treated with Both SH003 and DTX

**Figure 1:**
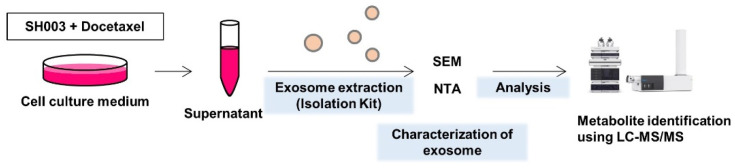
Workflow of the exosomal metabolite analysis in H460 lung cancer cells treated with SH003 and/or DTX.

To isolate exosomes secreted after combination therapy by SH003 and DTX, the culture supernatant from H460 lung cancer cells was collected, purified, and analyzed for altered metabolites (Figure 1).

**Figure 2:**
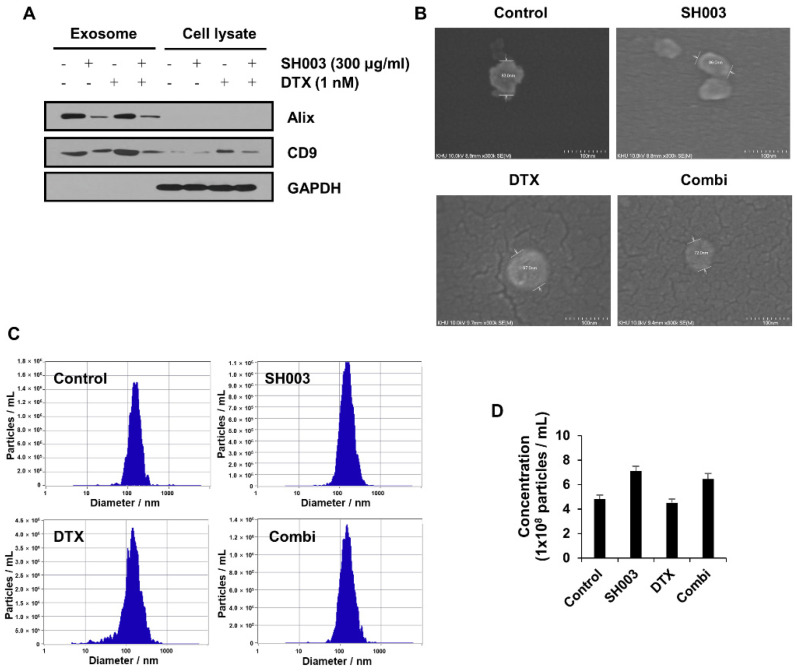
Verification of exosomes secreted by treatment of SH003 and DTX. (**A**) Exosomes were isolated using an extraction kit and suspended with PBS. The levels of exosomal and cellular proteins were analyzed by Western blotting. (**B**) SEM was used to examine the exosome morphology. The scale bar represents 100 nm. (**C**) The distribution of the diameter (nm) and the number of exosome particles were assessed by NTA. (**D**) The average concentration of exosomes.

The expression of exosomal markers Alix and CD9 [28] was enriched in the exosome fraction compared with that in the cell lysate (Figure 2A). SEM was used to examine the morphology and diameter of exosomes, which indicated that exosomes were round vesicles and uniform with a size of approximately 100 nm (Figure 2B). The exosome size and concentration distribution were assessed by NTA. The majority of particles had a diameter range of 145–165 nm (Figure 2C and Table 1) and the particle concentration showed no difference between drug treatment groups (Figure 2D). Thus, we established an exosome isolation method and found that the combinatorial treatment was not related to the number of exosomes secreted from cells.

### 3.2. Metabolome Analysis of Exosomes after Combinatorial Treatment

**Figure 3:**
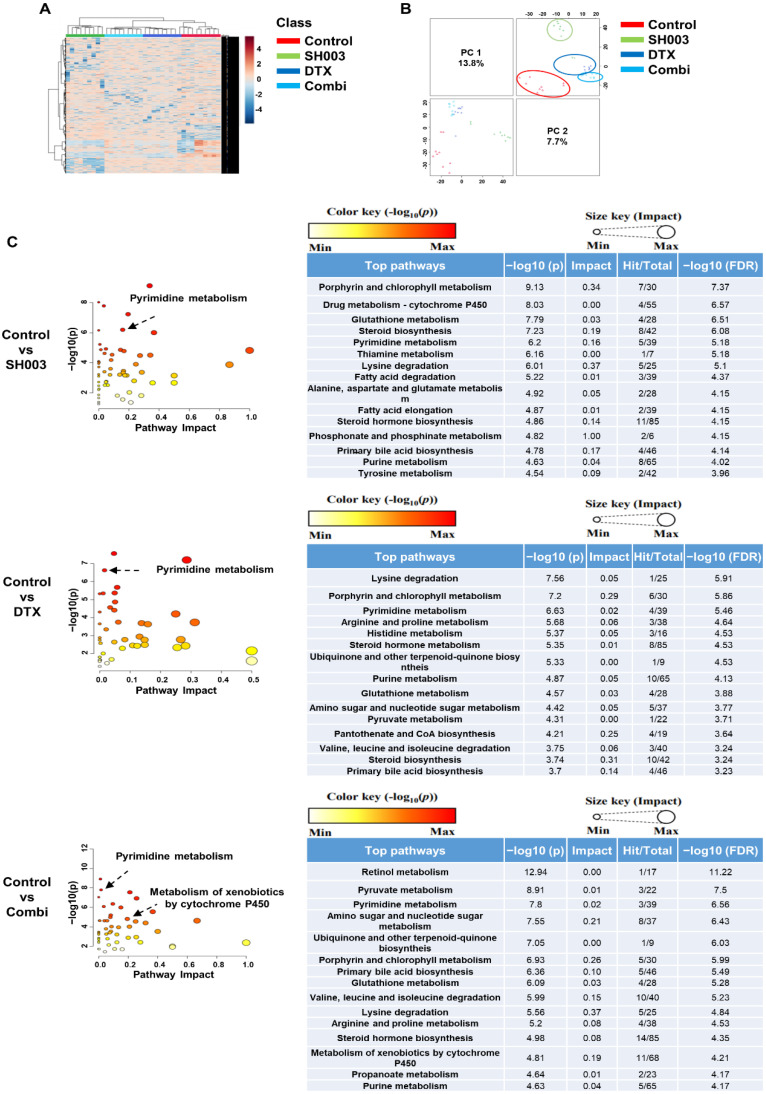
Exosome metabolite profiling. Purified exosomes were analyzed by LC-MS/MS. (**A**) HCA showed the clustered m/z features of exosomes in SH003, DTX, and combination groups. (**B**) PCA score plots showing the variation of clusters based on measured metabolites. (**C**) The pathway on significant metabolites identified after combined SH003 and DTX treatment was mapped from the KEGG database. Enrichment statistics and topology analysis of pathways were performed by MetaboAnalyst 5.0.

**Figure 4:**
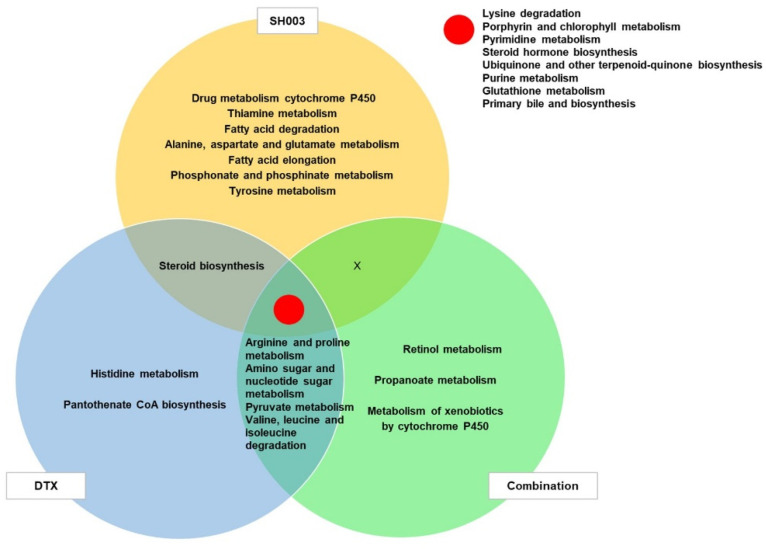
Venn diagram of overlapped pathways altered in exosomes among the three treatment groups. Pathways identified by KEGG analysis were classified by their *p*-value.

We identified what metabolites were influenced after combined treatment compared with SH003 or DTX alone by LC-MS/MS. The abundance levels of metabolites were transformed to the log2 value and normalized. To investigate the metabolite difference between groups, we first performed hierarchical cluster analysis (HCA) and principal component analysis (PCA) on the four groups. Each drug treatment showed a distinct clustering of metabolites (Figure 3A,B). To classify the characteristics of these metabolites, pathways corresponding to significant metabolites (*p* < 0.05) were mapped from a KEGG database, and pathway enrichment and topology were analyzed using MetaboAnalyst 5.0. As a result, we identified that six pathways, including retinol metabolism, pyrimidine metabolism, lysine degradation, arginine and proline metabolism, metabolism of xenobiotics by cytochrome P450, and propanoate metabolism, have a significant impact on combination treatment (Table 2). The results also revealed other metabolic features in response to SH003, DTX, and combined treatments (Figure 3C). Furthermore, a Venn diagram illustrated the distribution of metabolic pathways enriched in the treatment groups (Figure 4). We found pathways that overlapped among all treatment groups as follows: lysine degradation; porphyrin and chlorophyll metabolism; pyrimidine metabolism; steroid hormone biosynthesis; ubiquinone and other terpenoid-quinone biosynthesis; purine metabolism; glutathione metabolism; and primary bile and biosynthesis. However, three pathways, including retinol metabolism, metabolism of xenobiotics by cytochrome P450, and propanoate metabolism, were only mapped in combinatorially treated exosomes.

### 3.3. Identification of Metabolites Altered by Combinatorial Treatment

**Figure 5:**
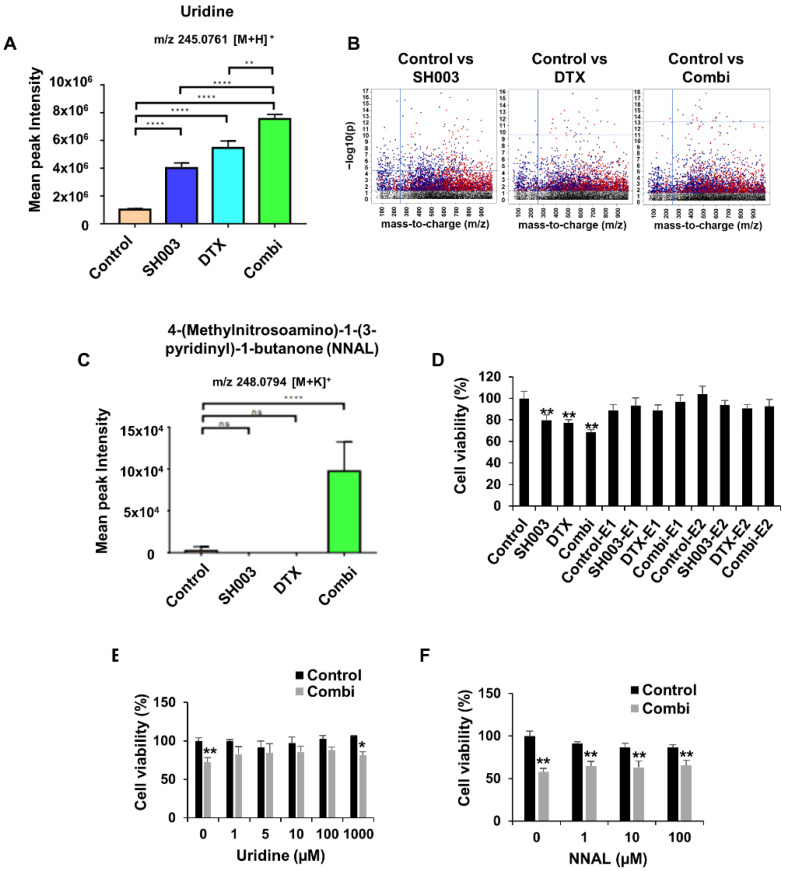
Major metabolites regulated by combined treatment. (**A**) The abundance levels of uridine. (**B**) Manhattan plot showing −log10(p) vs mass-to-charge (m/z) of metabolites. m/z feature above the dashed horizontal line meets the selection criteria. Red dots: lower in control group; dark blue dots: higher in control group. The blue dotted line represents the log *p*-value of uridine and blue line is arranged by 245.0761 m/z feature of uridine. (**C**) The abundance levels of NNAL. (**D**) Cells were treated with 20 and 40 μg/mL exosomes or SH003 and/or DTX for 24 h. Cell viability was analyzed by WST assays. E1: exosome 20 μg/mL; E2: exosome 40 μg/mL. (**E**,**F**) Cells were pre-treated with both SH003 and DTX for 1 h and then incubated with uridine or NNAL for 24 h. * *p* < 0.05, ** *p* < 0.01, and **** *p* < 0.0001 relative to the control by one-way ANOVA with Tukey’s post-hoc test.

Next, we investigated functional metabolites modulated by combined treatment. Of the metabolites shown in Table 2, we identified two metabolites, uridine and NNAL, which were significantly increased by the combined treatment (*p* < 0.05). Uridine is involved in pyrimidine metabolism, which was significantly altered in all groups (Figure 4), and plays an important role in the synthesis of glucose, lipids, and amino acids [29]. The level of uridine was increased by combinatorial treatment at a 7.4 ratio compared with that in the control, SH003, and DTX groups (Figure 5A). As shown in Figure 5B, the volcano plot also showed upregulation of uridine by drug exposure in all treated samples compared with that in the control. Furthermore, the NNAL metabolite belonging to the metabolism of xenobiotics by cytochrome P450 was enriched in only the combined group (Figure 4), showing a higher level of approximately 42-fold compared with that in the control (Figure 5C). Therefore, we determined whether exosomal metabolites secreted after combinatorial treatment were related to cell–cell communication for the death of lung cancer cells. When only exosomes secreted from drug-treated cells were treated in cells, it did not affect the cell viability (Figure 5D). These results suggested that the combination of SH003 and DTX led to cell death regardless of the exosome-mediated signal transduction. Additionally, neither uridine nor NNAL altered the effect of the combination on cell viability, showing no significant difference compared with the combined treatment (Figure 5E,F). Thus, we concluded that metabolites enriched in exosomes may be biomarkers to predict the therapeutic response for cancer suppression rather than improved drug sensitivity of combination treatment.

## 4. Discussion

Despite remarkable progress in diagnosis and therapeutic options for NSCLC patients, new cases and deaths of NSCLC have increased for both men and women from 1998 to 2021. Recently, exosomes have been used as a diagnostic marker for cancer and as a predictive marker for therapy [3]. Exosomes released from tumor cells reflect the hallmarks of cancer, and genetic materials and other substances within exosomes provide important information about the intracellular and intercellular signaling that occurs in cancer. Therefore, profiling exosome-carried molecules will help to better understand cancer treatments. Previous studies have shown that SH003 has anti-cancer properties and the potential for combination with DTX in NSCLC treatment. In this study, we identified markers that predicted the effect of combined treatment by analyzing the metabolic changes in exosomes induced by SH003, DTX, or both in vitro. Our results showed that metabolites and metabolic pathways as biomarkers indicated the treatment outcome of SH003-DTX combination-treated H460 cells.

Abnormal metabolic regulation for faster energy supply is considered as a hallmark of cancer. Therefore, to deal with complex cancer metabolism, metabolomics has been continuously increasing in cancer research [30]. Miyamoto et al. demonstrated the potential role of metabolites as cancer diagnostic markers by identifying altered metabolites in lung cancer patients by GC-TOF MS analysis [31]. In this study, the metabolites changed by SH003 and DTX were identified by LC-MS/MS. The screening results showed that “pyrimidine metabolism in cancer”, especially uridine, was a metabolite changed significantly by the combined treatment of H460 cells with SH003 and DTX. Uridine is involved in the synthesis of the building block, including proteins, nucleic acids, and lipids, for tumor cell proliferation via the pyrimidine salvage pathway. In terms of these activities associated with tumorigenesis, decreasing the intracellular uridine pool can be helpful for cancer proliferation-targeted therapy and increase the efficacy of anti-cancer drugs [32]. Uridine also inhibits side effects such as neurological deficits and myelotoxicity caused by pyrimidine metabolism-targeting drugs [33,34]. We assumed that the increased uridine in exosomes was associated with the synergistic effect, but both released exosome and uridine did not affect the inhibition of lung cancer growth. These results suggest that uridine is a putative biomarker to evaluate combination efficacy. In addition, based on the known protective effect of uridine on normal tissues, it is necessary to prove the possibility that the increased uridine of exosomes is involved in the effective cancer suppression mechanism of combination therapy through alleviation of toxicity to normal tissues.

In the metabolomics data, a metabolic change in xenobiotics by cytochrome P450 (CYP) was selectively induced only by the combined treatment. Among related metabolites, NNAL was significantly enhanced by the combined treatment (Figure 5C). NNAL is mostly known as an intermediate metabolite of nicotine, a xenobiotic, and metabolized by the CYP2A6 enzyme [35,36]. Because xenobiotics, which are recognized as foreign substances in cells, can potentially cause toxicity, they are absorbed, detoxified, and excreted by CYP enzymes to maintain a stable state within the cell [37]. However, CYP enzymes induce cell damage by inducing the biotransformation of toxic substances depending on their subtype. Therefore, the pharmacokinetics and pharmacodynamics of anti-cancer drugs are determined by the function of the CYP enzyme. Many studies have shown that NNAL is a metabolite detected as a carcinogen in lung cancer patients who smoke. However, the relationship of NNAL with the mechanism of anti-cancer drugs has not been elucidated. Our data showed that NNAL did not potentiate the cytotoxicity of the combined treatment (Figure 5F). Therefore, similar to uridine, we concluded that NNAL may be a response biomarker for combined treatment by SH003 and DTX. However, further studies are required to show how CYP enzymes and xenobiotic mechanisms are related to an effective response to SH003 and DTX.

Although the efficacy and molecular mechanism of SH003 have been well evaluated in non-clinical and clinical studies, further studies are required to find therapeutic biomarkers. Because changes in metabolites reflect the indispensable quality of drug dynamics in cells, this study revealed metabolites in exosomes as biomarkers to evaluate the effectiveness of combined treatment. Combined treatment with SH003 and DTX did not affect exosome production, but different levels of uridine and NNAL metabolites were found in released exosomes, showing potential as biomarkers for drug-efficacy evaluation. However, it is necessary to identify the metabolic pathways regulated by SH003 and DTX in cells to use these metabolites as biomarkers and to check the level of metabolites in blood, which do not interfere with combination therapy.

## 5. Conclusions

This study suggests exosomes as an indicator to evaluate the effectiveness of SH003 and DTX combination therapy rather than the function of exosomes as an inducer of cancer cell inhibition involved in cell–cell interactions. Therefore, metabolite analysis of exosomes can explore new biomarkers to evaluate concomitant efficacy. Additionally, analysis of altered metabolites will aid in the understanding of novel molecular mechanisms that inhibit cancer cells induced by SH003 with multidrug action properties.

## Figures and Tables

**Table 1:** The size distribution and concentration of nanoparticles by NTA.

	Peak Diameter (nm)	Particles/mL	%	Mean (nm)
Control	163.4	1.5 × 10^6^	51.2	158.5
	144.3	1.5 × 10^6^	46.1	
	303.8	1.5 × 10^5^	2.7	
SH003	153.8	1.2 × 10^6^	100	164.3
	145.5	4.2 × 10^6^	72.2	159.9
Docetaxel	114.2	3.4 × 10^6^	21.5	
	53.9	4.2 × 10^5^	6.3	
Combination *	147.9	1.3 × 10^6^	96.9	163
	339	1.9 × 10^5^	3.1	

* Combination indicates co-treatment of SH003 and docetaxel.

**Table 2:** The abundance level of metabolites identified in exosomes of combination- or single-treated cells.

			Control			SH003			DTX			Combi			T test (*p* Value)					
	mz	time	Mean	SD	SEM	Mean	SD	SEM	Mean	SD	SEM	Mean	SD	SEM	Control vs. SH003		Control vs. DTX		Control vs. Combi	
Retinol metabolism																				
beta-Carotene	575.3958	786.2536	2305	6914	2305	206,037	279,956	93,319	285,444	120,658	40,219	272,445	30,756	10,252	0.0294	*	0.0004	***	<0.0001	***
Pyrimidine metabolism																				
2′-Deoxy-5-hydroxymethylcytidine-5′-diphosphate	418.0422	769.836	122,100	55,140	18,380	96,258	151,995	50,665	24,520	50,510	16,837	0	0	0	0.0336	*	0.0063	**	<0.0001	***
2′-Deoxy-5-hydroxymethylcytidine-5′-triphosphate	535.9679	554.8825	206,112	85,006	28,335	164,458	316,409	105,470	94,471	204,050	68,017	220,157	165,718	55,239	0.0336	*	0.0063	**	0.1259	
Uridine	245.0761	316.7708	1,019,626	227,421	75,807	4,013,268	1,098,555	366,185	5,464,452	1,485,010	495,003	7,548,178	967,850	322,617	<0.0001	***	<0.0001	***	<0.0001	***
dUDP	371.0011	604.2127	67,650	51,578	17,193	58,477	118,058	39,353	131,200	55,963	18,654	107,978	16,564	5521	0.0063	**	0.0336	*	0.0063	**
dUMP	347.0017	350.6969	79,779	61,204	20,401	86,368	133,099	44,366	77,786	78,168	26,056	87,092	40,605	13,535	0.2882		0.9737		0.3517	
dUTP	506.9332	705.5207	13,140	30,905	10,302	33,207	99,622	33,207	124,109	81,905	27,302	40,246	60,053	20,018	0.7176		0.0019	**	0.848	
Lysine degradation																				
L-Lysine	129.1022	873.5476	5860	8855	2952	32,586	10,740	3580	32,740	43,308	14,436	24,262	6844	2281	0.0007	***	0.093		0.0007	***
N6-(L-1,3-Dicarboxypropyl)-L-lysine	277.1371	44.96933	105,655	20,446	6815	69,513	27,810	9270	34,041	19,798	6599	39,090	15,954	5318	0.0063	**	<0.0001	***	<0.0001	***
5-Phosphonooxy-L-lysine	281.0275	461.769	75,761	39,205	13,068	121,458	8788	2929	141,050	37,636	12,545	113,290	21,591	7197	0.0063	**	0.0336	*	0.0063	**
2-Oxoadipate	199.0014	888.6948	41,562	18,121	6040	157,442	66,139	22,046	62,238	26,272	8757	39,224	10,768	3589	0.0007	***	0.1259		0.9895	
N-Acetylputrescine	131.1173	361.3031	126,478	58,625	19,542	211,008	158,323	52,774	57,457	68,535	22,845	30,973	2945	981.6	0.1259		0.0336	*	0.0007	***
Glutaryl-CoA	882.1579	600.7987	145,656	107,450	35,817	140,440	226,181	75,394	260,080	116,159	38,720	57,769	85,988	28,663	0.1146		0.1259		0.1259	
Arginine and proline metabolism																				
4-Aminobutyraldehyde	88.07495	175.7108	143,178	56,241	18,747	53,268	110,275	36,758	173,923	81,752	27,251	127,881	78,918	26,306	0.0063	**	0.1259		0.9895	
4-Guanidinobutanoate	146.0915	55.58422	59,007	60,056	20,019	7716	23,148	7716	0	0	0	27,310	33,490	11,163	0.0007	***	<0.0001	***	0.1259	
Spermidine	146.1648	46.3017	170,411	52,614	17,538	200,397	57,593	19,198	119,336	62,308	20,769	73,007	72,973	24,324	0.0007	***	<0.0001	***	0.1259	
N-Acetylputrescine	113.1069	874.4732	52,133	45,246	15,082	7599	22,797	7599	9669	11,657	3886	65,659	63,978	21,326	0.0007	***	0.1259		0.3517	
L-Glutamate 5-semialdehyde	154.0467	17.7201	92,822	37,286	12,429	95,409	131,679	43,893	154,858	22,740	7580	100,759	44,569	14,856	0.1259		<0.0001	***	0.7301	
trans-3-Hydroxy-L-proline	154.0467	17.7201	92,822	37,286	12,429	95,409	131,679	43,893	154,858	22,740	7580	100,759	44,569	14,856	0.1259		<0.0001	***	0.7301	
cis-4-Hydroxy-D-proline	154.0467	17.7201	92,822	37,286	12,429	95,409	131,679	43,893	154,858	22,740	7580	100,759	44,569	14,856	0.1259		<0.0001	***	0.7301	
(R)-3-Amino-2-Methylpropanoate	86.06042	821.6563	132,513	42,946	14,315	31,0233	44,331	14,777	151,340	48,679	16,226	116,901	34,186	11,395	<0.0001	***	0.1259		0.7301	
Metabolism of xenobiotics by cytochrome P450																				
Benzo[a]pyrene-7,8-diol	269.0944	791.3047	201,542	121,100	40,367	77,150	144,628	48,209	97,513	116,385	38,795	140,782	114,059	38,020	0.029	*	0.1146		0.3517	
Benzo[a]pyrene-4,5-epoxide	269.0944	791.3047	201,542	121,100	40,367	77,150	144,628	48,209	97,513	116,385	38,795	140,782	114,059	38,020	0.029	*	0.1146		0.3517	
Benzo[a]pyrene-7,8-epoxide	269.0944	791.3047	201,542	121,100	40,367	77,150	144,628	48,209	97,513	116,385	38,795	140,782	114,059	38,020	0.029	*	0.1146		0.3517	
Benzo[a]pyrene-9,10-epoxide	269.0944	791.3047	201,542	121,100	40,367	77,150	144,628	48,209	97,513	116,385	38,795	140,782	114,059	38,020	0.029	*	0.1146		0.3517	
9-Hydroxylbenzo[a]pyrene	269.0944	791.3047	201,542	121,100	40,367	77,150	144,628	48,209	97,513	116,385	38,795	140,782	114,059	38,020	0.029	*	0.1146		0.3517	
1,1-Dichloroethylene	96.96057	203.2261	533,199	119,133	39,711	516,083	325,207	108,402	590,390	25,927	8642	472,406	45,756	15,252	0.7301		0.0336	*	0.1259	
1,1-Dichloroethylene epoxide	112.9561	887.2592	158,296	66,239	22,080	165,189	157,468	52,489	143,482	99,269	33,090	175,132	59,608	19,869	0.1259		0.7301		0.7301	
Chloroacetyl chloride	112.9561	887.2592	158,296	66,239	22,080	165,189	157,468	52,489	143,482	99,269	33,090	175,132	59,608	19,869	0.1259		0.7301		0.7301	
4,5-Dihydro-4-hydroxy-5-S-glutathionyl-benzo[a]pyrene	558.1676	787.7941	72,075	51,507	17,169	60,519	100,124	33,375	86,611	89,559	29,853	67,481	51,654	17,218	0.0336	*	0.3517		0.7301	
7,8-Dihydro-4-hydroxy-5-S-glutathionyl-benzo[a]pyrene	558.1676	787.7941	72,075	51,507	17,169	60,519	100,124	33,375	86,611	89,559	29,853	67,481	51,654	17,218	0.0336	*	0.3517		0.7301	
2,2-Dichloroacetaldehyde	112.9561	887.2592	158,296	66,239	22,080	165,189	157,468	52,489	143,482	99,269	33,090	175,132	59,608	19,869	0.1259		0.7301		0.7301	
(1S,2R)-Naphthalene 1,2-oxide	145.0652	50.46406	170,470	54,915	18,305	252,364	76,667	25,556	124,575	102,539	34,180	97,295	68,028	22,676	0.0336	*	0.0063	**	0.0063	**
(1R,2S)-Naphthalene 1,2-oxide	145.0652	50.46406	170,470	54,915	18,305	252,364	76,667	25,556	124,575	102,539	34,180	97,295	68,028	22,676	0.0336	*	0.0063	**	0.0063	**
4-(Methylnitrosamino)-1-(3-pyridyl)-1-butanol(NNAL)	248.0794	317.7357	2321	4919	1640	0	0	0	0	0	0	97,397	35,068	11,689	0.4706		0.4706		<0.0001	***
1-(Methylnitrosoamino)-4-(3-pyridinyl)-1,4-butanediol	226.1207	367.8325	52,277	21,209	7070	77,463	3554	1185	50,549	6684	2526	48,744	5941	1980	0.0063	**	0.6476		0.7301	
alpha-[3-[(Hydroxymethyl)nitrosoamino]propyl]-3-pyridinemethanol	226.1207	367.8325	52,277	21,209	7070	77,463	3554	1185	50,549	6684	2526	48,744	5941	1980	0.0063	**	0.6476		0.7301	
Propanoate metabolism																				
(S)-methylmalonate semialdehyde	140.9936	875.0135	129,827	64,967	21,656	91,637	105,217	35,072	128,205	62,406	20,802	114,891	59,161	19,720	0.1259		0.9895		0.9895	
2-Oxobutanoate	140.9936	875.0135	129,827	64,967	21,656	91,637	105,217	35,072	128,205	62,406	20,802	114,891	59,161	19,720	0.1259		0.9895		0.9895	
Thiamin diphosphate	408.0386	125.1114	3220	9660	3220	47,367	38,566	12,855	122,786	71,811	23,937	119,663	67,467	22,489	0.009	**	0.004	**	0.0007	***
Acetoacetyl-CoA	890.0926	600.5392	80,604	62,873	20,958	389,992	161,620	53,873	234,119	101,068	33,689	218,945	59,623	19,874	0.0007	***	0.0007	***	<0.0001	***

* indicates < 0.05; ** indicates < 0.01; *** indicates < 0.001.

## Data Availability

The data presented in this study are available in the article.
